# The role of food transfers in wild golden lion tamarins (*Leontopithecus rosalia)*: Support for the informational and nutritional hypothesis

**DOI:** 10.1007/s10329-020-00835-0

**Published:** 2020-06-24

**Authors:** Camille A. Troisi, William J. E. Hoppitt, Carlos R. Ruiz-Miranda, Kevin N. Laland

**Affiliations:** 1 School of Biology, University St Andrews, St Andrews, KY16 9TH UK; 2 Present Address: School of Biological, Earth and Environmental Sciences, University College Cork, Cork, Ireland; 3 Royal Holloway, London, UK; 4 Universidade Estadual do Norte Fluminense, Campos dos Goytacazes, Brazil

**Keywords:** Golden lion tamarins, Social learning, Teaching, Food transfer, Informational hypothesis

## Abstract

Callitrichidae is a unique primate family not only in terms of the large number of food transfers to infants but also for the prevalence of transfers that are initiated by the adults. It has been hypothesized that, as well as provisioning infants, callitrichid food transfers might function to teach the receiver what food types to eat. If food provisioning has a teaching function, we would expect successful food transfers to be more likely with food types that are novel to the juveniles. We would also expect juveniles to learn about foods from those transfers. We introduced different types of food (some familiar, some novel) to wild groups of golden lion tamarins (*Leontopithecus rosalia*). While novel foods were not more successfully transferred than familiar food in the experiment, transfers were more successful (i.e., the receiver obtained food) when the donor had previous experience with that food. Moreover, we found evidence suggesting that food transfers influenced the future foraging choices of juveniles. Our findings are consistent with the first and third criteria of the functional definition of teaching, which requires that tutors (the adults) modify their behavior in the presence of a naïve individual (a juvenile), and that the naïve individual learns from the modified behavior of the demonstrator. Our findings are also consistent with the provisioning function of food transfer. Social learning seems to play an important role in the development of young tamarins’ foraging preferences.

**Electronic supplementary material:**

The online version of this article (10.1007/s10329-020-00835-0) contains supplementary material, which is available to authorized users.

## Introduction

Food provisioning is a form of parental care. Adult–juvenile food transfers can have short-term benefits for the recipient as they allow the offspring to receive nutrients and energy that it might not have had otherwise (the nutritional hypothesis of food provisioning) (G.R. Brown et al. 2004). However, food provisioning can have additional longer-term benefits. For instance, while transferring food, adults can also transfer information about the food items’ quality or processing techniques. If information or skills are also transferred, this could help young reach nutritional independency by allowing them to learn about diet breadth and/or foraging skills of the species (a.k.a. the informational hypothesis) (G.R. Brown et al. 2004). The two hypotheses are not mutually exclusive, as receivers of a food transfer will typically obtain nutritional value from the food item when they acquire information about its palatability or quality (G.R. Brown et al. 2004).

The informational hypothesis is linked to teaching behavior. For a behavior to be considered teaching in a functional sense, an individual needs to (1) modify its behavior in the presence of a naïve observer; (2) this modification needs to come at a cost or at least no direct benefit; and (3) the naïve observer needs to learn a skill or information either earlier in life or that it would not have otherwise (Caro and Hauser 1992). Previous studies have investigated teaching in the context of learning about novel foods in hens (*Gallus gallus domesticus*: Nicol and Pope 1996), and white-tailed ptarmigans (*Lagopus leucurus*: Allen and Clarke 2005; Clarke 2010). Despite considerable interest in animal teaching, there are presently only four species that fulfil all of Caro and Hausers’ (1992) three criteria: tandem-running ants (*Temnothorax albipennis*: Franks and Richardson 2006), meerkats (*Suricata suricatta*: Thornton and McAuliffe 2006), pied babblers (*Turdoides bicolor*: Raihani and Ridley 2008), and superb fairywrens (*Malarus cyaneus*: Colombelli-Négrel et al. 2012; Kleindorfer et al. 2014a, b). Of these, only the case of teaching in meerkat is in a foraging context, where wild meerkats teach their pups how to handle prey, but probably not what to eat (Thornton and McAuliffe 2006). Teaching would be expected to evolve when relatedness between teachers and pupils is high (e.g., parent–offspring), when the opportunities or costs of learning from inadvertent social learning or asocial learning are high (e.g., solitary hunting), and when the information or skill transferred is frequent enough in the population to be possessed by the teacher (Fogarty et al. 2011; Hoppitt et al. 2008; Thornton and Raihani 2008, 2010).

Across the primate order, infants spend a lot of time feeding with group members, yet direct food transfer is quite uncommon (Brown et al. 2004; Rapaport and Brown 2008). In marked contrast, Callitrichidae is a primate family unique not only for the extensive transfer of food to infants but also for the prevalence of active giving initiated by adults and sub-adults (Brown et al. 2004; Feistner and McGrew 1989). There has been an extensive amount of work on adult-to-juveniles food transfers amongst callitrichids in captivity (Brown et al. 2004). In common marmosets (*Callithrix jaccus*), infants beg more for novel food compared to familiar food, suggesting that food transfers play a role in transmitting information about this novel food (Voelkl et al. 2006), however, they are not more likely to obtain them, suggesting that adults may sample the food before passing it on (Brown et al. 2005). Anecdotal evidence in common marmosets suggests that learning may also be promoted by the mother, where the mother changed her behavior according to the presence and age of the young (Dell’Mour et al. 2009). The mother solved the tasks, consumed less food and consumed it later, when she was foraging with older offspring or alone, compared to younger offspring, potentially allowing younger offspring with the opportunity to learn what to eat and how to forage (Dell’Mour et al. 2009). Captive golden-headed lion tamarins (*Leontopithecus chrysomelas*) transfers occurred more often when the food was difficult to access than when it was not (Moura et al. 2010). However, when novel food was available, transfers decreased compared to familiar food (Moura and Langguth 1999). The authors suggest that this pattern was due to an avoidance of potentially toxic food, and that juveniles might learn what to eat or what not to eat this way. Cotton-top tamarins (*Saguinus oedipus*) avoid transferring food that had been adulterated by pepper (Snowdon and Boe 2003), with adults more likely to share food when their own motivation towards food is high, and juveniles begging more for preferred food than control food item (Feistner and Chamove 1986). Moreover, adults’ withdrawal from food transfer encouraged independent feeding in the young (Joyce and Snowdon 2007) and solving a foraging task (Humle and Snowdon 2008). Furthermore, in cottontop tamarins, adults show some behavioral scaffolding, and seem to monitor the progress of their young: they increase their refusal of food transfers following their offspring’s first success at the task (Humle and Snowdon 2008). Similarly, in golden lion tamarins (*Leontopithecus rosalia*), the rate of refusal to transfer is influenced by juvenile-independent foraging (Rapaport and Ruiz-Miranda 2006). These findings suggest that adults may be adjusting their food transfer behavior to accelerate juveniles’ independent feeding. However, this pattern is not observed in all callitrichids. For instance, food transfer in pied bare-faced tamarins (*Saguinus bicolor*) seemed to be influenced by changes in the infants’ rather than the adults’ behavior (Price and Feistner 2001).

Food transfers are often accompanied by food-associated calls. In captivity, infants are more likely to obtain food from an adult, when adults produced food calls (golden lion tamarins: Brown and Mack 1978; Ruiz-Miranda et al. 1999; cotton-top tamarins: Joyce and Snowdon 2007; Roush and Snowdon 2001). Those vocalizations may play an important role in directing the juveniles’ attention towards food, and may be a form of information donation about what food to include in the diet, on which substrate to focus, or how to communicate about food (Rapaport, 2011; Rapaport and Ruiz-Miranda 2002; Roush and Snowdon 2001; Troisi et al. 2018).

In golden lion tamarins, a species of Callitrichidae, adult–juvenile food transfers seem particularly important for the development and survival of the young as juveniles are dependent on others to receive their first solid foods, and initially receive most of their solid food from food transfers. A captive study found that golden lion tamarins still receive up to 90% of their solid food from others at 16 weeks of age (Hoage 1982), before gradually becoming independent foragers by 9 months of age.

Golden lion tamarins are an ideal species in which to study teaching, as they are cooperative breeders, meaning that the relatedness between putative teachers and pupils is high, and that the cost of putative teaching is shared amongst several individuals. They also hunt individually for relatively large prey, suggesting low opportunities for inadvertent social learning (Rapaport 1999). They have a broad diet, with ephemeral and patchily distributed food sources, creating a need during ontogeny to rapidly learn what food are good to eat (Dietz et al. 1997). Given their high reproductive turnover, and short maturation period, teaching could be a strategy to speed up the learning of essential foraging information, and reducing the burden of provisioning young by hastening the transition to independent foraging (Troisi et al. 2018). Moreover, there is already some evidence supporting teaching in golden lion tamarins in another foraging-related context. Rapaport (2011) and Rapaport and Ruiz-Miranda (2002) suggest that adults may use food-offering calls, a vocalization often emitted prior to food transfers, to indicate a substrate where the juveniles can find prey. Furthermore, although the evidence was rather weak and based on small sample sizes, Troisi et al. (2018) found evidence that juveniles can seemingly learn in which substrate to forage through attending to those food-offering calls.

In the wild, food items that are voluntarily transferred to juvenile golden lion tamarins are more likely to be vertebrate and invertebrate prey (Ruiz-Miranda et al. 1999) than fruits. In a captive study with lion tamarins, Price and Feistner (1993) found that when food items are more difficult to acquire for juveniles (out of reach), and when items are presented singly (rare) rather than all at once, food transfers from adults to young increase and so did the adult’s response to juvenile begging. The results from this captive study suggest that food transfers in lion tamarins allow juveniles to receive adequate amounts of food. In the wild, reintroduced animals were also found to transfer a high number of provisioned bananas, an easily obtained food, further supporting the nutritional hypothesis (Ruiz-Miranda et al. 1999). However, another study on captive golden lion tamarins by Rapaport (1999) found that novel foods (be they novel to all individuals or novel to the young but familiar to the adult) are transferred to juveniles more than familiar ones. This would support the informational hypothesis, since experience of novel food items will provide more valuable information to juveniles about which foods to eat. However, Price and Feistner (1993) found that although juveniles ate less of the novel food, this pattern was not compensated by an increased transfer of those foods from adults to juveniles. This second result suggests that food transfers are not used to transmit information to juveniles about what to include in their diets. However, it should be noted that in this study only one golden lion tamarin took part in the experiment, the other subjects being golden-headed lion tamarins and black lion tamarins (*Leontopithecus chrysopygus*).

The seemingly contradictory results between Rapaport (1999) and Price and Feistner (1993) could highlight the dual role of food transfers in golden lion tamarins depending on the juveniles’ age. In Price and Feistner’s (1993) study, the juveniles were younger (7–21 weeks) than in Rapaport’s (1999) study (13–37 weeks). Plausibly, young juveniles, who sustain a high growth rate, might primarily receive food that they would not be able to acquire otherwise (nutritional hypothesis), while older immature individuals might mainly receive food that they have not sampled yet (informational hypothesis). By changing their behavior (first criterion), adult golden lion tamarins might teach the young what to incorporate in their diet. However, it remains unknown whether food transfers in wild golden lion tamarins also constitute a case of teaching, by allowing juveniles to learn about their diet (third criterion).

The aim of this experiment was to examine whether golden lion tamarins teach their young what to eat using food transfers. We first wanted to see if Rapaport’s (1999) findings hold in the wild, i.e., whether adults modify their food transfer behavior in the presence of juveniles (first criterion of the teaching definition). If food transfers were mainly for nutritional purposes, we would expect that the food novelty has no impact on the pattern of transfers: either all food items are transferred equally, or the most nutritious food are preferentially transferred. If transfers serve mainly for an informational objective, then we would expect the probability of success of an attempted food transfer (i.e., proportion of attempts in which the recipient receives food) involving a food that is novel to the receiver to be higher than the probability of success for familiar food, thereupon giving the juvenile the opportunity to learn. We also further investigated the role of the donor and receiver in food-transfer patterns by examining the decision of juveniles to attempt a food transfer and the decision of adults to resist those food transfers. We then wanted to examine whether juveniles learn from the food transfers (the third criterion of the teaching definition). If juveniles learn from food transfers, then we would expect that their experience with food through food transfers is more important in predicting their future food choice than other experience with food (such as eating food independently). We do not quantify the cost of food transfers in our experiment, so as such we are unwilling to make any claims regarding criterion 2 of the teaching definition.

## Methods

### Design

We introduced different food types to groups of wild golden lion tamarins in their natural environment over two time periods (thereupon “phases”) to evaluate which factors would affect the transfer of food between individuals, particularly between adults and subadults (thereupon adults) and juveniles, and understand the role of transfers in future food choices. At the time of first exposure (January–February 2014), this provided the opportunity for adults to transfer food to juveniles and for juveniles to learn about the different food types, both independently, and from social interactions (first phase). This first phase was conducted during the second half of the wet season (Dietz et al. 1994). Seven months later (August–September 2014), just before the start of the wet season (Dietz et al. 1994), we assessed how previous experience with the different food types influenced juveniles’ food choice once they were independent foragers (second phase). During both phases, the experiment took place in times of food abundance.

### Subjects

We studied six readily accessible groups of wild golden lion tamarins that were habituated to regular human contact and consistently monitored, in Silva Jardim municipality, Rio de Janeiro, Brazil. Three groups were at the Poço das Antas Biological Reserve, and three groups were in a fragment of Atlantic forest at the Fazenda Afetiva-Jorge, Imbaú region. At the start of the experiment, 42 individuals from those six different groups participated in the experiment, including ten juveniles between 4 and 5 months old. Each study group had one or two juveniles (golden lion tamarins often give birth to twins). This age range was chosen because juveniles are still dependent on adults for provisioning, and is in line with previous captive studies (Price and Feistner 1993; Rapaport 1999). Group AF2 lost both juveniles during the first phase of the experiment (after four valid trials), and before the start of the second phase of the experiment group Alone lost one juvenile. Thus, although the analysis regarding food-transfer patterns in the first phase of the experiment includes both juveniles of group AF2 and group Alone (*N* = 10) as well as all the adults present, the analysis regarding learning in the second phase of the experiment does not include the three juveniles that disappeared and was carried out on *N* = 7 juveniles (in five groups). The juveniles’ choices in the second phase of the experiment were assessed when the juveniles had reached an age of 11–12 months and were no longer reliant on adults for foraging. More information on the subjects and study site can be found in Table S1 of the Electronic Supplementary Materials (ESM), and in Troisi et al. (2018).

### Apparatus

**Fig. 1:**
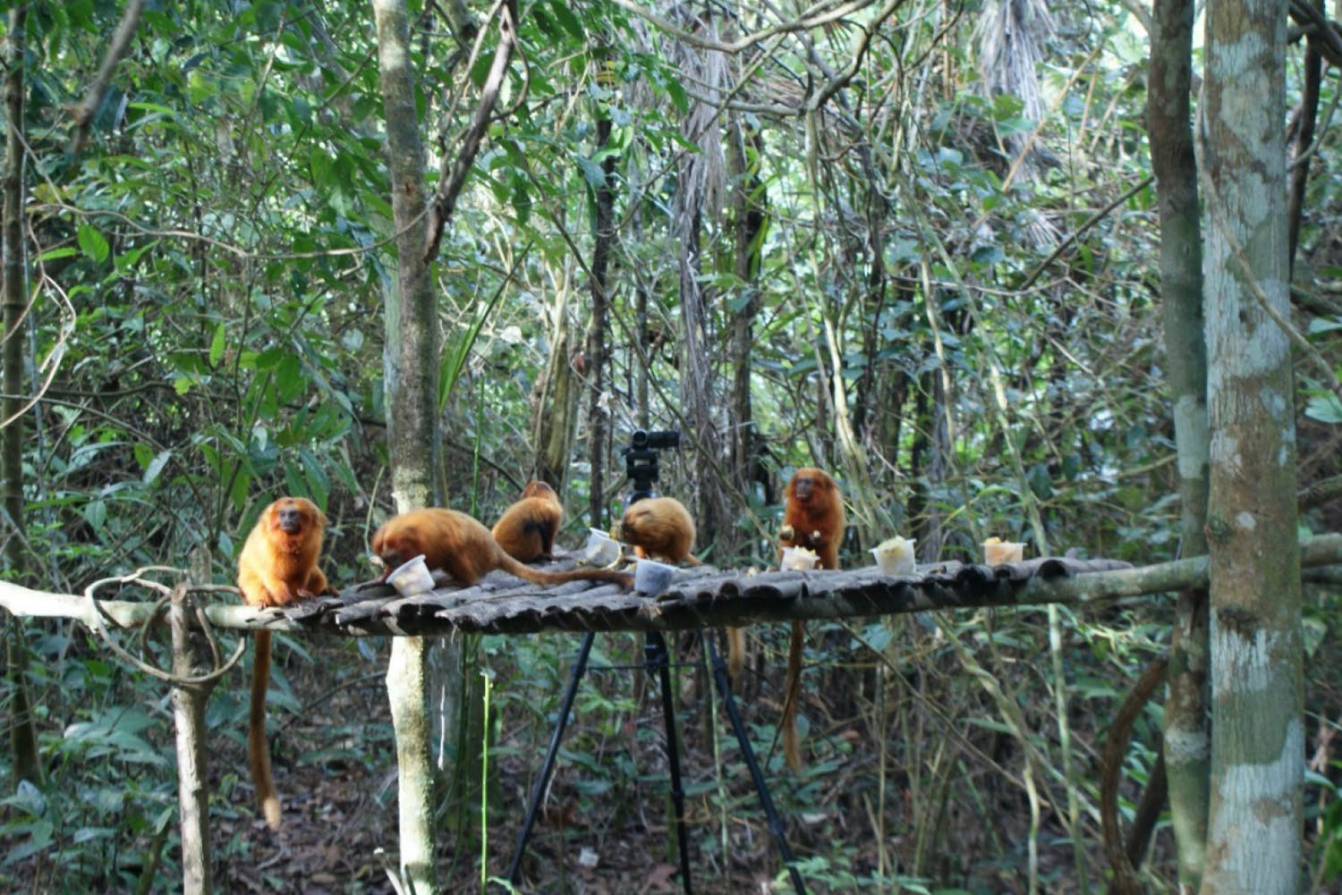
Seven pots containing different types of food attached to a platform during a trial to understand the role of food-transfers in golden lion tamarins at Poço das Antas and Affetiva, Fall 2014. One of the two cameras used to record trials can be seen in the background

Limited amounts of each food type were presented in separate, clear, plastic pots that were attached to a platform or to branches at human chest level (Fig. 1). The pots were approximately 7 cm in diameter and 5.5 cm in depth.

### Procedure

#### First phase

In the first phase, each group was exposed to five food options at the same time. These were: apple, banana, cricket, grape, and mealworm (see Table S2). Food options were arranged semi-randomly to ensure that most of the time the insect types were not adjacent to each other, and that when the trial did not occur on a platform (where the pots could be arranged in a circle) the familiar food had a fairly central place. Both insects and fruits were used to replicate the golden lion tamarins’ natural diet. Two types of food were provided: familiar and novel food. Familiar foods are food types that the golden lion tamarins in this population will have previously eaten prior to the start of this experiment, while novel foods are food types that the golden lion tamarins in this population have not eaten previously to the start of the experiment. Banana was a familiar food for all golden lion tamarins, while the other fruit options were novel. The novel foods were chosen based on the food used in captive studies with Callitrichids (Brown et al. 2005; Rapaport 1998; Vitale and Queyras 1997; Voelkl et al. 2006). The fruits were cut into small pieces (< 2 cm), to fill the pots, and insects were small enough so that several insects could fill the pots. Individuals had no access to those novel foods outside of the experimental context. Despite using food types regularly used in captivity, the dehydrated insects were rarely eaten in our experiment, and were classified as novel foods. We provide more information about our choices of food in the ESM.

Each trial was conducted on a different day (Table S3). Groups were tested on their own, but trials were considered invalid if no juveniles were present, or if individuals were present on the foraging platform for less than 80 s in total. Trials were repeated until five valid trials had been completed per group so that each group would have approximately the same opportunities, and all trials (valid and invalid) were filmed and used for later analysis. Trials continued until all individuals had left (average length of trial for both phases: 11 min 10 s, standard deviation: 9 min 54 s). The dates of all valid trials can be found in Table S3 of the ESM.

#### Second phase

For the second phase, five trials were conducted for each group deploying the same criteria as in the first phase. This time, two new novel foods were added to the experiment (papaya and pear) bringing the total food options to seven. We added two food types the juveniles had no prior experience with to allow us to test for an effect of individual experience on foraging choice in the second phase. In group AF3, for one of the valid trials, the camera was covered with dew, so we were unable to extract from the video recording most of the data for that trial. We therefore conducted an extra trial for that group and included all trials in the analysis. The dates of all valid trials can be found in Table S3 of the ESM.

### Video analysis

We extracted data from videos using the software package VideoLAN Client (VLC). We recorded behavior patterns (Table 1) as states in Microsoft Excel but treated them as discrete events in the analysis. Ten percent of the data were double-coded and the inter-observer reliability was found to be high (*r* = 0.95, *p* < 2.2e−16).

**Table 1:** Definitions of the dependent variables used for the analysis looking at the patterns of food transfers and their consequences on juvenile golden lion tamarins’ foraging choices

Behavior	Definition
Exploration	The individual shows interest in the food by orientating its face towards the food and being close enough to sniff it (no physical contact, but close proximity) or handle the food (physical contact) without putting the items in its mouth
Individual eating	Ingestion of food obtained from the pots, or from just outside the pots (platform, branch, or ground)
Food transfer	Any interaction between two individuals involving a food item. This includes an individual offering the food item it has to another individual, but also events where one individual attempts to obtain a food item from another individual, either by emitting vocalizations or by reaching out an arm in that direction. A successful food transfer is a food transfer in which the receiver obtained some food from the donor For each food transfer, we recorded the identity of both receiver and donor individuals, whether the donor resisted the transfer and whether or not the transfer was successful
Successful food transfer	The receiver obtained part or the entire food item. We note that successful transfer did not necessarily lead to eating, as on some, very rare, occasions the receiver obtained food from the donor, but then dropped or discarded it. Unsuccessful food transfer occurred when a food transfer was attempted but the receiver did not receive any parts of the food
Social eating	Ingestion event that resulted from a successful food transfer
Resistance	During a food transfer, we noted whether the donor turned away from the receiver, held on to the item while the receiver was trying to get it, or ran away

There is a wide range of food transfer types that have been recognized in callitrichids, from a donor actively sharing food, passively sharing it, food being eaten out of the hand of the donor or food being stolen (Feistner and Price 1990; Hoage 1982; Rapaport 1998). Previous studies have also distinguished different types of food transfers but analyzed them together. Because of the rarity of food transfers where the donor actively transferred food to the receiver in our dataset, we first describe findings with those active “giving” transfers before statistically analyzing all types of food transfers and looking at more subtle behavioral cues such as juveniles’ attempts and adults’ resistance to transfers.

### Statistical analysis

We carried out all analysis using R version 3.6.1 (R Core Team 2019). In order to determine the relative importance of the predictor variables in each model, we used an information-theoretic approach with model averaging as described in Grueber et al. (2011) using the dredge function from the *MuMIn* package (Barton 2019). We calculated the relative degree of support for each variable using the Akaike Information Criterion corrected for small sample sizes (AICc) (Burnham and Anderson 2002). The Results section reports the model-averaged parameter estimates, their unconditional standard errors (incorporating model selection uncertainty), and their 95% confidence intervals. We also report the corresponding back-transformed effect on odds and their 95% confidence intervals (Galipaud et al. 2014). See the ESM section “4. Statistical analysis: model averaging methods” for more details on model averaging procedures.

#### First criterion: modification of behavior

##### Probability of success of a food transfer

To analyze the probability of success in a food transfer, we specified a global model using a generalized linear mixed model (GLMM) with a binomial error structure using the *lme4* package (Bates et al. 2015). We included both receiver and donor individual as random effects. See the ESM section “5. Statistical analysis: treatment of the random effects” for more details on the random effects in our models. We tested the dataset to ensure that the assumptions were not violated. We checked for overdispersion using the dispersion_glmer function in the *blmeco* package (Korner-Nievergelt et al. 2015).

Four main explanatory variables were used. The first three variables were dependent on the food option, *F*, involved in a given food transfer. We were first and foremost interested in whether the type of food (novel or familiar) would impact the probability of success, and thus looked at the effect of food familiarity, defined as whether *F* was familiar (banana), or not, to the tamarins prior to the experiment’s start (binary variable). We were also interested in whether individuals updated their knowledge on the food types during the course of the experiment. Accordingly, we included an option-specific success variable for both the receiver and donor individuals, where ‘option’ refers to the different food options available to the golden lion tamarins. Option-specific success calculates the number of each food item previously ingested at any given time for any given individual. Donor option-specific success was the amount of *F* (number of food items) the donor individual had consumed during the experiment prior to the food transfer in question, whereas receiver option-specific success was the equivalent variable for the potential receiver. These variables were included to test whether there was a possible familiarization with the food items as the experiment went on. We also included variables giving characteristics of individuals: donor age and receiver age were binary variables representing whether the donor and potential receiver respectively were a juvenile or not, and, donor sex and receiver sex gave the sex of each individual involved in the food transfer.

We then refit the set of models replacing the continuous variables donor option-specific success and receiver option-specific success with corresponding binary variables, indicating whether donor option-specific success > *0* and whether receiver option-specific success > 0. This was to allow for the possibility (suggested by data exploration) that consuming a single food item of type *F* may be sufficient for the food to become familiar to a tamarin, or that individuals are neophobic and might require at least some experience with the experimental setup before adopting their usual behavior.

We want to highlight that food familiarity was determined before the experiment, and therefore novel foods (apples and grapes) were considered novel to all, while familiar foods (bananas) were considered familiar to all. Option-specific success on the other hand relates to the number of ingestions of a particular food type to each individual, which changes throughout the experiment, and is therefore dependent on each individual’s experience.

##### Probability of attempting a food transfer

We then examined whether the patterns of food transfers observed were mainly due to the receivers, and particularly whether wild juveniles attempted to obtain more novel food than familiar food. To analyze the probability of juveniles attempting a food transfer from adults, we used a GLMM with a binomial error structure using the *lme4* package (Bates et al. 2015).

For each combination of potential receiver, potential donor, food option, and receiver option-specific success, we calculated the number of opportunities for attempting a food transfer, defined as an event in which a potential donor was ingesting a food item and the potential receiver was present at the time of the event. We then calculated the number of these events in which a food transfer was attempted to obtain the dependent variable for the analysis. There were no opportunities of food transfers between adults and juveniles for mealworms, hence its absence as a food type in this analysis. There were also only five opportunities for crickets, and no food transfers, so we excluded them from the analysis. In this analysis, we included receiver option-specific success as a binary variable, since we found no effect of receiver option-specific success as a continuous variable in the previous analysis. Similarly, we included the variable of food option rather than food familiarity, as we found no effect of food familiarity as a binary variable in the previous analysis. Random effects were included as above (potential receiver and potential donor individual).

##### Probability of resistance (during a transfer)

We then examined the involvement of the donor in determining the probability that a food transfer would be successful. As a proxy of the donor’s preference for keeping versus giving up food items, we used resistance during a food transfer. To analyze the probability of resistance, we specified a global model using a GLMM with a binomial error structure using the *lme4* package (Bates et al. 2015). Similar to the previous analysis, data were restricted to transfers in which potential receivers were juveniles and potential donors were adults. For three food transfers, the presence of resistance was unknown, so we excluded those cases from the analysis. The presence of resistance in a transfer was modeled as a function of food option, previous receiver, and donor option-specific success (as binary) and sex of both the donor and receiver. Random effects were included as above (receiver and donor individual).

#### Third criterion: learning

The final aspect of teaching behavior that we wanted to explore in a food transfer context was whether or not juveniles learn about the transferred food as a result of the adult’s modified behavior (third criterion of teaching definition). We modeled juveniles’ food choices in the second phase of the experiment (when they were independent foragers ~ 11 months old) as a function of their prior social and asocial experience (during the first phase, when juveniles were ~ 4 months old). The dependent variable was the number of times each food item was ingested independently (not eating a food after obtaining it from a transfer), by each juvenile, for each food type, during the second phase of the experiment. The independent variables were the number of times during the first phase of the experiment where each food type had been eaten following a food transfer (social eating), which was the main factor of interest, as well as the number of times each food type have been eaten independently (individual eating) and exploration. Individual was included as a random effect.

For two food types (papaya and pear), there was no previous experience, and for two other food types (cricket and mealworms), there was little previous experience, leading to the possibility of zero-inflated data. We therefore compared candidate models with different error structure, and with and without accounting for zero-inflation, based on their overdispersion parameter, and their AIC (see Table S4 in the ESM, for AICs and overdispersion parameters of candidate models, and Figure S1 for the parameter estimates of each model). Each model was fitted using the glmmadmb function in the *glmmADMB* package (Fournier et al. 2012; Skaug et al. 2016). The best global model that showed no overdispersion was a negative binomial zero-inflated model (family = “nbinom”, log link) and was used for further analysis.

### Data availability

The datasets analyzed during the current study and the R Code used to analyze them are available on the Open Science Framework repository: https://osf.io/cpkvy/ (DOI: 10.17605/OSF.IO/CPKVY).

## Results

### First criterion: modified behavior

#### Qualitative analysis of food transfers

During the first phase of this study, 233 attempted food transfers were made by 32 adults golden lion tamarins and ten juveniles, from six different groups. Attempted food transfers comprised 7% of all foraging-related behavior (eating and exploring food). Forty-eight percent of those transfers were successful, meaning the recipient obtained food (111/233); 51% of the attempted food transfers were from an adult to a juvenile (119/233), whereas the other 49% were transfers from adult to adult (80/233), juvenile to adult (24/233), and juvenile to juvenile (10/233). From those adult–juvenile attempted transfers, 53% were successful (63/119). There were a total of 1243 successful ingestion events, so golden lion tamarins in the first phase of the experiment obtained 9% of ingested food items from food transfers. Seventy percent of the successful food transfers were made with novel food, and 67% when only juveniles were recipients and adults were donors.

Out of those 233 attempted transfers, only 12 had a donor active and initiating the transfer (5%). All of those donor-initiated transfers were successful, and nine of those interactions were transfers of grapes (*N* = 7) or apples (*N* = 2), which were both novel foods, while three were of bananas. In all of those transfers, the donor was an adult or subadult, but the age of the receivers varied. Nine receivers of donor-initiated transfers were juveniles, and three were subadults. All of the subadult receivers were females. Because all of the donor-initiated transfers were successful, it was not possible to analyze whether the transfers of novel food were more successful than transfers of familiar food. Instead, we ran an analysis over all of the food transfers, and then separately looked at the role of receivers through their attempts at obtaining food from other individuals and the role of donors through resistance.

#### Probability of succeeding in a food transfer

There was little support for an effect of the number of previous option-specific ingestions by the potential recipient (support = 13%, effect size = − 0.002; 95% CI = − 0.02, 0.01, Table S4, S5) or the potential donor (support = 9%, effect size = 0.001; 95% CI = − 0.01, 0.01, Table S5, S6). However, this analysis assumes that the odds of a successful food transfer will be a linear function of the previous number of successes. An alternative possibility is that a single ingestion of a novel food item is enough for a tamarin to become familiar with a food type, and thus decrease the odds of success, without further ingestion events having an effect.

**Fig. 2:**
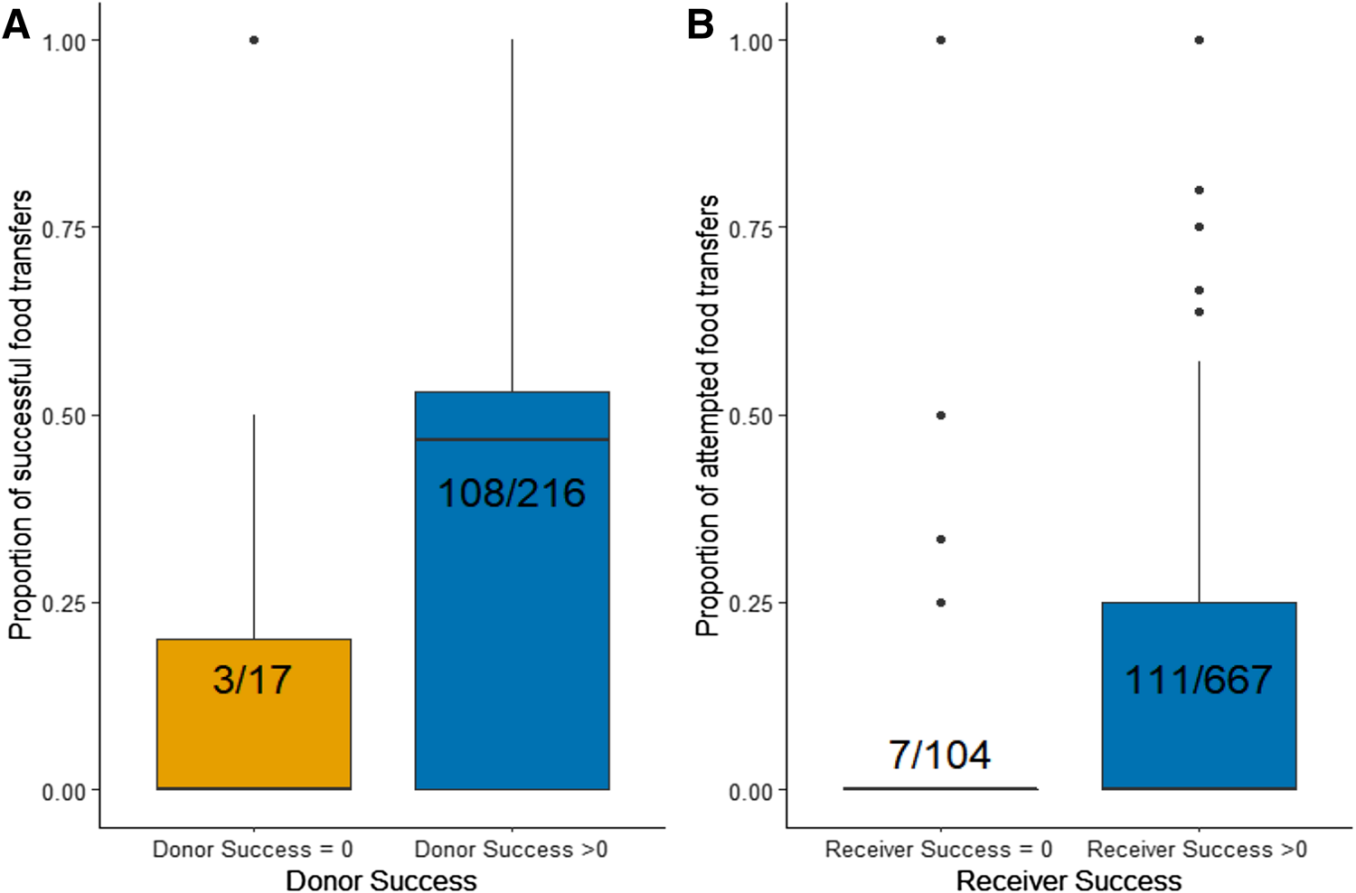
**a** Boxplot showing the effect of *donor option-specific success* (as binary) on the proportion of success of food transfers, averaged by individuals. **b** Boxplot showing the effect of *receiver option-specific success* (as binary) on the proportion of attempted food transfers, averaged by individual (**b** only includes food transfers with juveniles as recipients, and adults as donors). Data collected at Poço das Antas and Affetiva in January–February 2014

We therefore tested for an effect of option-specific success as a binary variable (success = 0 versus success > 0) on both the potential donor (donor option-specific success) and receiver (receiver option-specific success) of the transfer. Donor option-specific success > 0 came out as an important variable in predicting the success of a transfer. There was evidence of a strong effect of the donor having ingested a food item at least once, with the odds of success for an attempted food transfer being higher when the donor had ingested a food type at least once than when the donor has never ingested that type of food, suggesting that a single ingestion event is sufficient for a potential donor to treat a food type as familiar (Table 2, Fig. 2a). On the other hand, there was little support for an effect of receiver option-specific success as a binary variable (Table 2), which suggests that one exposure to the food item or experimental context does not change the receiver’s behavior. Similar to the previous analysis, we found little evidence of an effect of food familiarity on the probability of success of a food transfer (Table 2, S5, S6, S7). There was also little evidence of an effect of the age of the potential recipient, the age of the potential donor, or of the sex of the potential recipient or donor (Table 2).

**Table 2:** Results of the generalized linear mixed model looking at the probability of food transfer success

Variable	Sum of weights	Model averaged estimate (± unconditional SE)	95% CI	Back-transformed effect on odds of success	Back-transformed unconditional 95% CI
Intercept		− 1.40 (± 0.71)	− 2.79, − 0.02	0.25 baseline odds of success	0.06, 0.98
Donor option-specific success > 0	**1.00**	**1.81 (± 0.70)**	**0.43, 3.18**	**6.08** × **(success > 0/ success = 0)**	**1.54, 23.99**
Donor age	0.78	− 0.61 (± 0.51)	− 1.60, 0.39	0.54 × (juveniles/adults)	0.20, 1.47
Receiver option-specific success > 0	0.54	− 0.31 (± 0.41)	− 1.11, 0.49	0.73x (success > 0/ success = 0)	0.33, 1.63
Food familiarity	0.51	− 0.22 (± 0.31)	− 0.82, 0.37	0.80 × (familiar/novel)	0.44, 1.46
Receiver sex	0.19	− 0.05 (± 0.16)	− 0.36, 0.27	0.95x (females/males)	0.70, 1.30
Receiver age	0.17	− 0.03 (± 0.14)	− 0.25, 0.31	1.03 × (juveniles/adults)	0.78, 1.37
Donor sex	0.04	0.01 (± 0.07)	− 0.13, 0.15	1.01 × (females/males)	0.88, 1.16

The table shows the relative importance (sum of Akaike weights), estimates, unconditional standard errors, back-transformed effect on odds of success and their confidence intervals for parameters included in the top models. Includes donor option-specific success and receiver option-specific success as binary variables, food familiarity, donor and receiver age, and donor and receiver sex as fixed effects. Individuals were included as random effects. Entries in bold indicate *p* < 0.05. Data from Poço das Antas and Affetiva, January–February 2014

Both when option-specific success was continuous and binary, we found little evidence of a difference in success among donors, yet we cannot rule out a large effect either (Table S8).

#### Probability of attempting a food transfer

We then investigated the probability of juveniles attempting a food transfer from an adult when that individual was consuming food. This was based on 771 opportunities for ten potential juvenile receivers from six groups. Only receiver option-specific success as a binary variable seems to predict the probability of attempting a food transfer (Table 3, Table S9). In fact, when a juvenile has already ingested a specific type of food, it is more likely to attempt to obtain it than when it has never ingested it (Table 3; Fig. 2b). We found little evidence of a difference in attempting a food transfer towards different donors, yet we cannot rule out a large effect either (Table S8).

**Table 3:** Results of the generalized linear mixed model looking at the probability of juvenile golden lion tamarins attempting a food transfer from adults

Variable	Sum of weights		Model-averaged estimate (± unconditional SE)	95% CI	Back-transformed effect on odds of attempts	Back-transformed unconditional 95% CI
Intercept			− 2.59 (± 0.52)	− 3.61, − 1.56	0.08 baseline odds of attempt	0.03, 0.21
Receiver option-specific success > 0	**1.00**		**0.93 (± 0.42)**	**0.12, 1.74**	**2.54** × **(success > 0/ success = 0)**	**1.12, 5.72**
Receiver sex (female)	0.26		− 0.12 (± 0.35)	− 0.80, 0.56	0.89 × (female/male)	0.45, 1.74
Food option (baseline = Apple)	0.24	Banana	0.10 (± 0.22)	− 0.33, 0.54	1.11 × (banana/apple)	0.72, 1.11
		Grape	0.03 (± 0.15)	− 0.26, 0.33	1.03 × (grape/apple)	0.77, 1.39

The table shows the relative importance (sum of Akaike weights), estimates, unconditional standard errors, back-transformed effect on odds of success and their confidence intervals for parameters included in the top models. Individuals were included as random effects. Entries in bold indicate *p* < 0.05. Data from Poço das Antas and Affetiva, January–February 2014

#### Probability of resistance (during a transfer)

We then investigated the probability of resistance in attempted food transfers between adults and juveniles. Sixty-two percent of those transfers were resisted by the donor (72/116; Fig. 2c); 58% of attempted food transfers with resistance failed (42/72), while only 27% of attempted food transfers without resistance failed (12/44).

Analysis for the probability of resistance during a transfer was based on 116 food transfers of ten juvenile receivers from six groups. No variable had a particular importance in predicting resistance to a food transfer (Table 4, Table S10). Hence, adults seemed equally likely to resist attempted food transfers by juveniles regardless of the food option, and also equally likely to resist when they have already had an experience with the food option compared to when they have not. However, we found evidence of a difference in resistance among donors, as well as large effects (Table S8).

**Table 4:** Results of the generalized linear mixed model looking at the probability of adults resisting food transfers towards juveniles

Variable	Sum of weights		Model-averaged estimate (± unconditional SE)	95% CI	Back-transformed effect on odds of success	Back-transformed unconditional 95% CI
Intercept			1.30 (± 1.44)	− 1.52, 4.13	3.69 baseline odds of resistance	0.22, 61.87
Receiver option-specific success > 0	0.53		0.49 (± 0.66)	− 0.81, 1.80	1.64 × (success > 0/ success = 0)	0.45, 6.03
Donor option-specific success > 0	0.46		− 0.96(± 1.48)	− 3.86, 1.93	0.38 × (success > 0/ success = 0)	0.02, 6.92
Food option (baseline = Apple)	0.41	Banana	0.28 (± 0.54)	− 0.78, 1.33	1.32 × (banana/apple)	0.46, 3.79
		Grape	− 0.26 (± 0.52)	− 1.28, 0.75	0.77 × (grape/apple)	0.28. 2.12
Receiver sex	0.38		0.26 (± 0.46)	− 0.65, 1.16	1.29 × (females/males)	0.52, 3.21

The table shows the relative importance (sum of Akaike weights), estimates, unconditional standard errors, back-transformed effect on odds of success and their confidence intervals for parameters included in the top models. Individuals were included as random effects. Data from Poço das Antas and Affetiva, January–February 2014

### Third criterion: learning

Eating a food obtained from a food transfer (social eating) had a significant influence on the number of food items chosen by juveniles in the second phase of the experiment, and was the main predictor of juveniles’ future foraging decisions (Table 5, Table S11). In fact, eating a specific food type obtained from a food transfer made juveniles substantially more likely to eat more of this food (Table 5, Table S11).

**Table 5:** Results of the generalized linear mixed model looking at the number of eating events for each food type once juvenile golden lion tamarins were independent foragers

Variable	Sum of weights	Model-averaged estimate (± unconditional SE)	95% CI	Back-transformed effect on odds of attempts	Back-transformed unconditional 95% CI
Intercept		1.36 (± 0.35)	0.69, 2.04	3.92 baseline odds of attempt	1.99, 7.72
Exploration	0.55	0.03 (± 0.03)	− 0.03, 0.09	1.03 × per exploration events	0.97, 1.09
Social eating	**1.00**	**0.35 (± 0.16)**	**0.04, 0.66**	**1.42 × per social eating events**	**1.04, 1.94**
Individual eating	0.45	0.03 (± 0.04)	− 0.04, 0.11	1.03 × per individual eating events	0.96, 1.11

The table shows the relative importance (sum of Akaike weights), estimates, unconditional standard errors, back-transformed effect on odds of success and their confidence intervals for parameters included in the top models. Individuals were included as random effects. Entries in bold indicate *p* < 0.05. Data from Poço das Antas and Affetiva, January–February and August–September 2014

## Discussion

### Food transfer patterns

Contrary to our prediction, and unlike Rapaport’s (1999) findings, we found little evidence that novel foods were more successfully transferred than familiar ones by golden lion tamarins in the wild, suggesting that donors do not modify their behavior in this way. Our results however suggest that whether the donor had previously ingested a type of food or not proved to be a good predictor of the probability of a food transfer being successful, indicating that the first feeding event of the donor is important. It is possible that a single ingestion event is sufficient for potential donors to treat a food option as familiar, but one exposure to the food option or experimental context does not change the receivers’ behavior. The donor might only need to experience the food and/or the experimental setup once before transferring food successfully. Thus, individual learning and habituation might be necessary before engaging in any social interactions. Thornton and Raihani (2010) suggest that evidence of teaching about novel food would be strengthened by evidence that donors are willing to incur the cost of sampling novel food before transferring it to receivers, to assess the food’s palatability. The fact that transfers are more successful when the donor has sampled the food option at least once is consistent with the possibility that potential golden lion tamarin donors need to sample the novel food to make sure it is palatable, before transferring it. This supports the informational hypothesis and the important role of the donor in the transfer. This is also consistent with the nutritional hypothesis, where donors transfer food that is palatable, with a provisioning function.

In some callitrichids, juveniles beg more for novel food then they do for familiar items (Brown et al. 2005; Voelkl et al. 2006). If, as a consequence of their begging behavior, juveniles would receive more novel food than familiar food then they would be the ones responsible for this pattern of transfers, thus weakening the case for teaching criterion (Feistner and Price 2000; Price and Feistner 2001). However, both Rapaport (1999) and Price and Feistner (1993) found that juveniles beg as much for novel as for familiar food. Thus, if juvenile golden lion tamarins obtain more novel food than familiar food, as Rapaport’s (1999) results suggest, the adults (donors) would be responsible for that pattern, and not the juveniles (receivers). In our experiment, we found that the food option did not affect juveniles’ attempts to obtain food from other individuals. However, they attempted more food transfers of a food option that they had previously ingested in the context of this experiment (Table 3). This could suggest that the juveniles require some short adaptation time to the experimental set up before engaging in social interactions such as food transfer, or that there are some short-term effects of the familiarity of the food. This is contrary to findings in captivity where Rapaport (1999) and Price and Feistner (1993) found that juveniles beg as much for novel and for familiar food. However, despite juveniles making more attempts to obtain food they have ingested before, receivers are not more successful in obtaining those foods (Table 2). Taken together, the results suggest that transfer patterns are more influenced by the donor’s behavior than the juveniles’ attempts.

We also found that adult donors were as likely to resist attempted food transfers from juveniles when they had had previous ingestion experience with the food option compared to when they had not. Donors were also as likely to resist food transfers of any food option. Thus, adults do not seem to use food transfers as a way to get rid of food that they have no previous experience with, which could have explained the observed pattern of successful food transfers. This also reflects the cost of giving up any food, and adults might only transfer food when there is a fitness return, whether it helps the juvenile nutritionally and/or whether it helps them learn.

Overall, results from the analysis of the food transfer patterns suggest that golden lion tamarins transfer more food when they have previous experience with it than food with which they have no experience. Juveniles are also more likely to attempt obtaining food that they have previous experience with, although they are not more successful at obtaining it, compared to food that they have no previous experience with. Moreover, the pattern of successful food transfers is not explained by adults attempting preferentially to keep food that they have previous experience of. We therefore have support for both the nutritional and informational hypothesis, where food transfers are more likely to be successful if the donor has ingested the food previously potentially to ensure that the receiver is ingesting palatable food, as well as learning about food palatability. Our data support the fact that food transfers can have several functions. Although it is possible that human provisioning might have influenced the tamarins natural behavior, the direct effect of provisioning on foraging abilities in golden lion tamarins is unknown (Stoinski and Beck 2004), and we are unsure of how this might have influenced our results. But in all the analysis, we do account for individual differences.

### Lack of evidence for teaching

Contrary to teaching predictions, we found little evidence of an effect of food familiarity on the success of food transfers. It should be noted that only the bananas were familiar to the donors. It is possible that the lack of evidence supporting the role of food familiarity on food transfer success could be specific to bananas vs. novel food rather than familiar food vs. novel food. Moreover, in marked contrast with previous literature, it should also be noted that food transfer of insects did not occur. This was because the insects we provided were actually rarely eaten by our study population (only seven times), probably due to the fact that they were dried, dehydrated, and dead. It is possible that this also explains the small number of active food transfers (only 12 cases), as active transfers are mainly of prey (Ruiz-Miranda et al. 1999). The fact that active donations between adults were so rare in our study suggests that food transfer is unlikely to be a form of teaching about what fruits to eat. Further studies should reflect the logistical and ethical ways of incorporating live insects that would appeal to the tamarins, which we were unable to do.

Contrary to teaching predictions, we found little evidence of an effect of whether the recipient has already tried the food on food transfer success. Given that all individuals arrive at the same time, adults might be more interested in the food themselves than monitoring what the juveniles have experienced. Unfortunately, given logistical considerations, we were unable to present food that was familiar to the adults but unfamiliar to the juveniles. Future studies should incorporate this third type of novelty, like Rapaport (1999) did, as this is the type of food to be expected to be most successfully transfer under a teaching explanation.

Surprisingly, there was little evidence that transfers to juveniles were more successful than transfers to adults, which would be expected under a teaching explanation (Table 2). It is possible that food transfers serve another function than providing food or information to juveniles. Adult–adult food transfers are infrequent in primates, but they could function to create or strengthen social bonds as seen in some avian species (e.g., Liévin-Bazin et al. 2019). This has also been suggested very recently to be the case in other tamarin species, including in a close relative of the golden lion tamarin: the golden-headed lion tamarin (Guerreiro Martins et al. 2019). Anecdotal evidence of food transfer used to form or strengthen social bonds is also present in golden lion tamarins (Troisi 2020). There are also previous reports of food transferred to pregnant females in golden lion tamarins (Ruiz-Miranda et al. 1999). In fact, in our data, all three of the donor-initiated food transfers made to adults were made to females. Although we do not know if those females were pregnant, this fits with the pattern of previous findings, suggesting that food transfers to adults could also have a nutritional role (Ruiz-Miranda et al. 1999). Finally, it is also possible that those successful adult–adult food transfers are a form of harassment-avoidance, where food is transferred because it is the least costly behavior. This could also be one explanation for the adult-to-juvenile food transfers, but we find this unlikely as the juveniles do not seem to be the drivers behind the food transfer patterns, and there is no particular variable explaining the probability of donors resisting the transfer. Overall, we found support for both the nutritional and information hypothesis. It is possible that food transfers fulfil several processes at the same time: transferring information to young, creating bonds with other adults, providing nutrition to pregnant females… which could explain why we do not observe food transfers being more successful when the recipients are juveniles compared to when they are adult.

### Social learning

We found evidence that food transfers promote juvenile learning about which food to eat and do so more than individual eating events. Those long-term effects of food transfers support the informational hypothesis of food transfers in wild golden lion tamarins. Previous work has found that similarly, chickens and white-tailed ptarmigan chicks show a preference for food that has been demonstrated by their parent (Allen and Clarke 2005; Nicol 2004). In primates, social influences on the foraging behavior of juveniles is very prominent, particularly in great apes and callitrichids (Rapaport and Brown 2008). Social influences span from social facilitation when eating novel food in capuchins (Addessi and Visalberghi 2001; Visalberghi and Fragaszy 1995), scrounging in marmosets (Caldwell and Whiten 2003), co-feeding in chimpanzees (Ueno and Matsuzawa 2005), to conformity biases in vervets (van de Waal et al. 2013). In callitrichids, social facilitation plays an important role in novel food acceptance (Vitale and Queyras 1997; Voelkl et al. 2006; Yamamoto and Lopes 2004), but not success at solving a task (Moscovice and Snowdon 2006). In our experiment, the acceptance of novel food may not only be related to tasting the specific food item, or obtaining it from another individual, but to the facilitation provided by the presence of conspecifics eating any food at all. However, given that our experiment took place in a group setting, most of the foraging-related behavior (eating, exploring, food transfers) took place with other individuals around. We are therefore not able to examine the effect that social facilitation might have on the acceptance of novel food. Instead, we find a direct effect of food transfers, suggesting that the association between a particular food type and an interaction with a conspecific is important in influencing future independent food choices of juveniles.

Previous research found that previous social experience such as food-offering calls seem to facilitate juvenile learning of the availability of food at a substrate (Troisi et al. 2018). In this experiment, we further demonstrate the importance of social learning in this species in a new context: food transfers during early development are important for juveniles to learn which food to eat, once they are independent. Another study in primates previously found that food transfers influenced future dietary choices: infant common marmosets show a strong preference for food that had been obtained from others compared to food that they had experienced independently (van Bergen 2004). Our study suggests that this is also occurring in other callitrichids and in the wild.

Our results suggest that food transfers allow juveniles to acquire information about their diet, but previous work in captivity suggests that food transfers may play different functions depending on the age of the juveniles (Price and Feistner 1993; Rapaport 1999). Transfers to younger golden lion tamarin juveniles seem to follow the nutritional hypothesis, while transfers to slightly older juveniles seem to follow the informational hypothesis (Price and Feistner 1993; Rapaport 1999). Golden lion tamarins have been found to change the context in which they use food-offering calls in the wild (Rapaport 2011), and calls emitted to older juveniles promote learning of the substrate in which they should forage (Troisi et al. 2018). Future work should investigate whether the function of food transfers according to age varies in the wild as well. A study of food transfers in meerkats found that adults teach young how to manipulate prey (Thornton and McAuliffe 2006). Given that golden lion tamarins mainly transfer prey to their young, it would be interesting to examine whether juveniles also learn how to manipulate insect prey from food transfers as they get older. Overall, more work is needed to better understand the role of food transfers across the development of young in the wild, and this could be done by examining how food transfers at different time points and of different items vary with age.

## Conclusions

After examining the food transfer patterns of golden lion tamarins in the wild, contrary to our predictions, we did not find that transfers of novel food were more successful than transfers of familiar food, but we did find that transfers were more successful when the donor had already had experience ingesting a particular type of food. This suggests that donors might need to be knowledgeable about the food palatability before transferring it, which is consistent with both the informational hypothesis and a teaching function to food transfer (i.e., the first criterion). This is also consistent with the nutritional hypothesis. Potential receivers had little influence in the transfer pattern, but juveniles would beg more for food that they are more familiar with (from having ingested it at least once before), although they are not more successful at obtaining those foods.

Our study went on to look at whether juveniles learned what food to eat from the food transfer. We found evidence that food transfers were an important predictor of juvenile’s future foraging choices, which is again consistent with the information hypothesis and the third criterion of the teaching definition. Although we have some support for teaching, our data are also inconsistent with some of the teaching predictions, leaving open the possibility that food transfers evolved because of the twin provisioning and informational benefits.

Overall, there is little clear evidence for teaching in the context of food transfers for golden lion tamarins, but it cannot be ruled out. Social learning, on the other hand, seems to play an important role in the development of young golden lion tamarins’ foraging preferences and foraging searches (Troisi et al. 2018). Social learning can have crucial consequences for the survival of individuals and populations (Brakes et al. 2019). Despite recent successful efforts to increase population numbers of golden lion tamarins, they remain an endangered species (Ruiz-Miranda et al. 2019). Given recent findings, it seems essential to integrate their reliance on social learning in conservation practices.

## Electronic supplementary material

Below is the link to the electronic supplementary material.

Supplementary file1 (DOCX 62 kb)

Supplementary file2 (CSV 694 kb)

Supplementary file3 (CSV 1 kb)

Supplementary file4 (CSV 9 kb)

Supplementary file5 (R 20 kb)

Supplementary file6 (CSV 1 kb)
