# Supplementary material for: The role of food transfers in wild golden lion tamarins (Leontopithecus rosalia): Support for the informational and nutritional hypothesis
Source: Primates. 2020 Jun 24;62(1):207–21. doi: 10.1007/s10329-020-00835-0 (PMC7813722; doi:10.1007/s10329-020-00835-0)
Supplement: Supplementary file 1 — Supplementary file1 (DOCX 62 kb) [file 10329_2020_835_MOESM1_ESM.docx]

**ESM**

1. **Subjects**

Table S1: List of individuals that took part in the experiment. Ad = adult; Sub = subadult; Juv = juvenile; inf = infant; ABS = absent. Note that data of infants were not analysed.

| Locality | Group | Individual | Sex | Date of birth | Age during the first phase | Age during the second phase | Present in the first phase | Present in the second phase | Start date of the first phase | Start date of the second phase |
| --- | --- | --- | --- | --- | --- | --- | --- | --- | --- | --- |
| Poco | BO2 | 1284 | M | — | Ad | Ad | ✓ | ✓ | 5/02/2014 | 27/08/2014 |
| Poco | BO2 | 1278 | F | — | Ad | Ad | ✓ | ✓ | 5/02/2014 | 27/08/2014 |
| Poco | BO2 | 1342 | M | 10/12 | Sub | Ad | ✓ | ✓ | 5/02/2014 | 27/08/2014 |
| Poco | BO2 | 1343 | M | 10/12 | Sub | Ad | ✓ | ✓ | 5/02/2014 | 27/08/2014 |
| Poco | BO2 | 1351 | M | 02/13 | Sub | Ad | ✓ | ✓ | 5/02/2014 | 27/08/2014 |
| Poco | BO2 | 1352 | F | 02/13 | Sub | Ad | ✓ | ✓ | 5/02/2014 | 27/08/2014 |
| Poco | BO2 | 1353 | M | 09/13 | Juv | Sub | ✓ | ✓ | 5/02/2014 | 27/08/2014 |
| Poco | BO2 | 1354 | F | 09/13 | Juv | Sub | ✓ | ✓ | 5/02/2014 | 27/08/2014 |
| Poco | AF | FA3 | M | 10/09 | Ad | Ad | ✓ | ✓ | 10/02/2014 | 27/08/2014 |
| Poco | AF | AF13 | F | 12/08 | Ad | Ad | ✓ | ✓ | 10/02/2014 | 27/08/2014 |
| Poco | AF | AF19 | M | 10/11 | Ad | ABS | ✓ |  | 10/02/2014 | 27/08/2014 |
| Poco | AF | AF20 | F | 12/11 | Ad | Ad | ✓ | ✓ | 10/02/2014 | 27/08/2014 |
| Poco | AF | AF35 | M | 12/12 | Sub | Ad | ✓ | ✓ | 10/02/2014 | 27/08/2014 |
| Poco | AF | AF27 | F | 10/13 | Juv | Sub | ✓ | ✓ | 10/02/2014 | 27/08/2014 |
| Poco | Alone | 1303 | M | — | Ad | Ad | ✓ | ✓ | 17/02/2014 | 30/08/2014 |
| Poco | Alone | 1313 | F | 11/09 | Ad | ABS | ✓ |  | 17/02/2014 | 30/08/2014 |
| Poco | Alone | 1360 | F | — | ABS | Ad |  | ✓ | 17/02/2014 | 30/08/2014 |
| Poco | Alone | 1355 | M | 10/13 | Juv | Sub | ✓ | ✓ | 17/02/2014 | 30/08/2014 |
| Poco | Alone | 1356 | M | 10/13 | Juv | ABS | ✓ |  | 17/02/2014 | 30/08/2014 |
| Afetiva | AF3 | PT8 | M | 10/01 | Ad | Ad | ✓ | ✓ | 30/01/2014 | 28/08/2014 |
| Afetiva | AF3 | SP18 | M | 10/09 | Ad | Ad | ✓ | ✓ | 30/01/2014 | 28/08/2014 |
| Afetiva | AF3 | FA2 | F | 12/08 | Ad | Ad | ✓ | ✓ | 30/01/2014 | 28/08/2014 |
| Afetiva | AF3 | FP1 | M | 12/12 | Sub | ABS | ✓ |  | 30/01/2014 | 28/08/2014 |
| Afetiva | AF3 | FP2 | F | 12/12 | Sub | Ad | ✓ | ✓ | 30/01/2014 | 28/08/2014 |
| Afetiva | AF3 | FP3 | F | 09/13 | Juv | Sub | ✓ | ✓ | 30/01/2014 | 28/08/2014 |
| Afetiva | AF3 | FP4 | M | 09/13 | Juv | Sub | ✓ | ✓ | 30/01/2014 | 28/08/2014 |
| Afetiva | Super | SP16 | F | 11/08 | Ad | Ad | ✓ | ✓ | 6/02/2014 | 28/08/2014 |
| Afetiva | Super | FA4 | M | 10/09 | Ad | Ad | ✓ | ✓ | 6/02/2014 | 28/08/2014 |
| Afetiva | Super | SP20 | M | 11/11 | Ad | Ad | ✓ | ✓ | 6/02/2014 | 28/08/2014 |
| Afetiva | Super | SP23 | F | 12/12 | Sub | Ad | ✓ | ✓ | 6/02/2014 | 28/08/2014 |
| Afetiva | Super | SP24 | F | 12/12 | Sub | Ad | ✓ | ✓ | 6/02/2014 | 28/08/2014 |
| Afetiva | Super | SP25 | F | 12/12 | Sub | ABS | ✓ |  | 6/02/2014 | 28/08/2014 |
| Afetiva | Super | SP26 | F | 09/13 | Juv | Sub | ✓ | ✓ | 6/02/2014 | 28/08/2014 |
| Afetiva | Super | SP27 | F | 02/14 | Inf | Juv | ✓ | ✓ | 6/02/2014 | 28/08/2014 |
| Afetiva | Super | SP28 | F | 02/14 | Inf | Juv | ✓ | ✓ | 6/02/2014 | 28/08/2014 |
| Afetiva | AF2 | SP6 | M | 10/06 | Ad | Ad | ✓ | ✓ | 30/01/2014 | 28/08/2014 |
| Afetiva | AF2 | AF4 | F | 10/04 | Ad | Ad | ✓ | ✓ | 30/01/2014 | 28/08/2014 |
| Afetiva | AF2 | BE1 | M | 01/02 | Ad | Ad | ✓ | ✓ | 30/01/2014 | 28/08/2014 |
| Afetiva | AF2 | FA6 | F | 10/10 | Ad | Ad | ✓ | ✓ | 30/01/2014 | 28/08/2014 |
| Afetiva | AF2 | FA8 | M | 10/11 | Ad | Ad | ✓ | ✓ | 30/01/2014 | 28/08/2014 |
| Afetiva | AF2 | FA10 | M | 10/12 | Sub | Ad | ✓ | ✓ | 30/01/2014 | 28/08/2014 |
| Afetiva | AF2 | FA11 | M | 10/12 | Sub | Ad | ✓ | ✓ | 30/01/2014 | 28/08/2014 |
| Afetiva | AF2 | FA12 | F | 10/12 | Sub | Ad | ✓ | ✓ | 30/01/2014 | 28/08/2014 |
| Afetiva | AF2 | FA13 | M | 09/13 | Juv | ABS | ✓ |  | 30/01/2014 | 28/08/2014 |
| Afetiva | AF2 | FA14 | F | 09/13 | Juv | ABS | ✓ |  | 30/01/2014 | 28/08/2014 |
| Afetiva | AF2 | FA15 | F | 02/14 | Inf | Juv | ✓ | ✓ | 30/01/2014 | 28/08/2014 |
| Afetiva | AF2 | FA16 | F | 02/14 | Inf | Juv | ✓ | ✓ | 30/01/2014 | 28/08/2014 |

1. **Further information about the food items selected for the experiment**

The fruits were considered of lower nutritious value than the insects (see Table S2). Insects were dehydrated and from a local supplier (Nutrinsectat). Crickets were used because they are part of the golden lion tamarins’ diet in the wild, and were considered to be familiar prior to the experiment, and highly nutritious. Hence, the design was to have two familiar food options, one of lower nutritional value (banana) and one of higher nutritional value (cricket), and three novel food options, two of lower nutritional value (apples and grapes), and one of higher nutritional value (mealworms). Two different fruits were used as novel options to account for differences in texture, and potentially in preferences. This design would have allowed to tease apart whether golden lion tamarins transfer more readily highly nutritious food, or more readily novel food, or a combination of both. However, because the wild golden lion tamarins in the experiment did not readily feed on the dehydrated insects (only 7 times), we reclassified the crickets as novel for the analysis. Although this left only one familiar food, there still was variety in the nutritional values and textures of the novel foods.

Table S2: Nutritional values of the food options. – denotes that no information was found

| Food option (per 100g) | Calories | Fat (g) | Carbohydrates (g) | Proteins (g) |
| --- | --- | --- | --- | --- |
| Apple | 52 | 0.2 | 13.8 | 0.3 |
| Banana | 89 | 0.3 | 22.8 | 1.1 |
| Cricket | 120 | 5.5 | 5.1 | 12.9 |
| Grape | 67 | 0.4 | 17.2 | 0.6 |
| Mealworm | 206 | 13 | - | - |
| Papaya | 39 | 0.1 | 9.8 | 0.6 |
| Pear | 58 | 0.1 | 15.5 | 0.4 |

1. **Dates of valid trials**

Table S3: Dates on which each valid trial of the first and second phase of the experiment took place for each group

| First Phase | | | | | |
| --- | --- | --- | --- | --- | --- |
| Group | Trial 1 | Trial 2 | Trial 3 | Trial 4 | Trial 5 |
| BO2 | 05/02/14 | 13/02/14 | 14/02/14 | 09/03/14 | 12/03/14 |
| AF | 10/02/14 | 12/02/14 | 13/02/14 | 27/02/14 | 08/03/14 |
| Alone | 17/02/14 | 27/02/14 | 09/03/14 | 11/03/14 | 12/03/14 |
| AF3 | 30/01/14 | 31/02/14 | 16/02/14 | 21/02/14 | 23/02/14 |
| Super | 06/02/14 | 07/02/14 | 11/02/14 | 22/02/14 | 23/02/14 |
| AF2 | 30/01/14 | 01/02/14 | 03/02/14 | 04/02/14 | 06/02/14 |
| Second Phase | | | | | |
| BO2 | 27/08/14 | 30/08/14 | 31/08/14 | 06/09/14 | 07/09/14 |
| AF | 27/08/14 | 30/08/14 | 02/09/14 | 03/09/14 | 06/09/14 |
| Alone | 30/08/14 | 31/08/14 | 02/09/14 | 03/09/14 | 07/09/14 |
| AF3 | 28/08/14 | 29/08/14 | 01/09/14 | 04/09/14 | 08/09/14 |
| Super | 28/08/14 | 08/09/14 | 11/09/14 | 17/09/14 | 18/09/14 |

1. **Statistical analysis: model averaging methods**

We used the information-theoretic approach with model averaging as it allows us to compare several competing models (i.e. hypotheses) simultaneously and make inferences based on the weighted support for several models (Grueber et al., 2011). Model averaging also allows one to reduce model uncertainty, compared to stepwise methods of model selection (Grueber et al., 2011).

With this approach, a series of candidate models was generated, each representing a biological hypothesis. Hence, instead of testing each hypothesis independently using null hypothesis significance testing, we examined several hypotheses at the same time. The AIC is an index that takes into account the likelihood of the model as well as the number of parameters in that model (through parsimony), and ranges between zero and one (Nakagawa & Cuthill, 2007). By using AICs, issues related to conventional p-values, such as an arbitrary threshold, are avoided (Grueber et al., 2011). Moreover, by penalising the model for the number of parameters, this approach minimised the number of falsely positive predictors that were included in each model (Waite & Campbell, 2006). The model with the smallest AIC is supposed to retain all of the important predictors (Nakagawa & Cuthill, 2007). However if the first ranked model’s weight is close to that of other model(s), there is uncertainty about which model is the best (Burnham & Anderson, 2002; Grueber et al., 2011). In this case, interpretation should rely on a set of models, rather than just the best model (Burnham & Anderson, 2002; Symonds & Moussalli, 2011). Hence following Grueber et al.’s (2011) methodology, the set of “top” models was defined by taking the best model (the model with the lowest AICc value) and all the other models that were within two AICc units of the best model (Burnham & Anderson, 2002). Models within two AICc of the top model were considered to be as informative as the top model (Burnham & Anderson, 2002; Symonds & Moussalli, 2011). Here we used the full-model averaging approach, where the inference is based on all models in the candidate set, because of the high model uncertainty observed (when the best AICc is not strongly weighted) (Symonds & Moussalli, 2011). With the top models, the averaged parameter estimates for each predictor variable as well as their relative importance, were computed. The relative importance is calculated by summing the Akaike weights of all models where the predictor variable in question is present. Akaike weights represent the probability of a given model to being the best model compared to other models in the subset (Burnham & Anderson, 2002). The relative importance of a variable can therefore be thought of as the probability that this particular variable is part of the best model (Symonds & Moussalli, 2011). However, it is not the probability that the variable has a statistical effect (Galipaud, Gillingham, David, & Dechaume-Moncharmont, 2014). On top of the relative importance of the predictors present in the top models (those within two AICc units of the best model), the result section reports the parameter estimates, their standard errors, and their 95% confidence intervals as well as the back-transformed effect on odds and their 95% confidence intervals (Galipaud et al., 2014). Standard errors are unconditional, meaning that they incorporate model selection uncertainty.

1. **Statistical analysis: treatment of the random effects**

When analysing the probability of success of a food transfer, we initially used nested random effects of *receiver* and *donor individual* within *groups*, but the effect of *group* was estimated to be very small (variance = 0) and did not have any significant impact (p=1), so we dropped *group* as a nested random effect from the model to aid convergence of the model optimisation. We therefore included only both *receiver* and *donor* individual as random effects. However, this model still had singularity issues, so we ran the same model with only *receiver* *individual* as a random effect, with only *donor individual* as a random effect, and no random effects. The results for all four models were similar, so we report the model with both *receiver* and *donor individual* as random effects.

Similar to the analysis concerning the probability of success of a food transfer, when we analysed the probability of resistance during a transfer, the global model with both *receiver* and *donor individual* as random effects had problems converging, so we ran the same model with only *receiver* *individual* as a random effect, with only *donor individual* as a random effect, and no random effects. All models (no random effect, *individual receiver* as random effect, *individual donor* as random effect) showed similar results to the initial analysis in that no support for any of the explanatory variables was found. Therefore, in the results section we only report the results of the analysis including both random effects.

1. **Statistical analysis: learning models with different error structure**

Table S4: Table showing the full models with different model family and zero-inflation, their AIC, and the overdispersion parameter. In the main text, we model average the model in bold (nbin, ZINB), rather than the model with lowest AIC (nbin1, ZINB), as the model with the lowest AIC shows overdispersion

| Model family and zero inflated | dAIC | Df | Overdispersion parameter |
| --- | --- | --- | --- |
| ZINB1: Family: nbin1; ZINB | 0.0 | 7 | 4.04 |
| **ZINB: Family: nbin; ZINB** | **7.0** | **7** | **1.10** |
| Negbin1: Family: nbin1; no ZINB | 9.8 | 6 | 18.84 |
| Negbin: Family: nbin; no ZINB | 10.8 | 6 | 0.64 |
| ZIP: Family: poisson; ZIP | 151.2 | 6 | 454.71 |
| Poisson: Family: poisson; no ZIP | 288.4 | 5 | 587.74 |


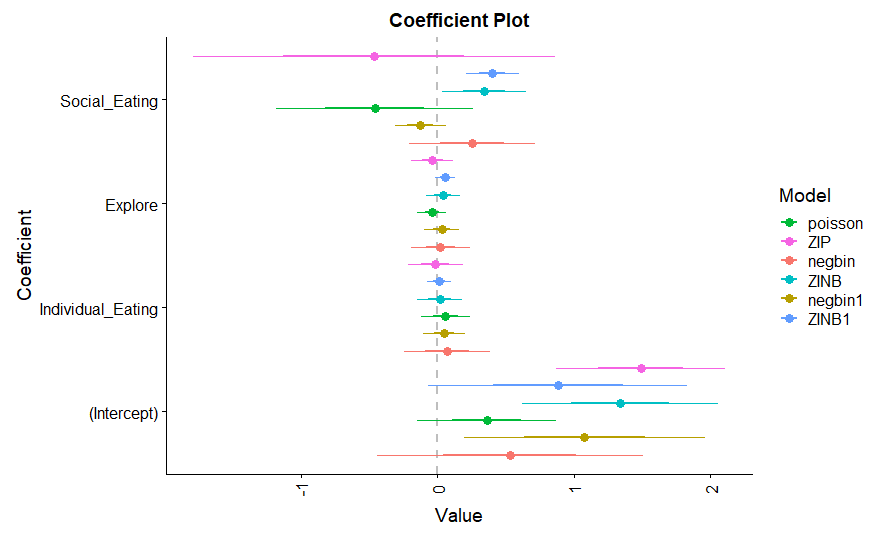


**Fig S1** Figure showing parameter estimates for each of the models shown in Table S4

1. **Results - First criterion: modified behaviour**
   1. Probability of succeeding in a food transfer

When investigating the probability of success of all food transfers, the top models fitted are shown in Table S5 and results of model averaging are in Table S6 of the ESM. There was little support for an effect of *food familiarity* on the probability of success of a food transfer (effect size = -0.12; 95% C.I. = -0.59, 0.36) with the odds of success for an attempted food transfer involving familiar food being 0.89 times lower (95% U.C.I. =0.55, 1.43) than attempted food transfers involving novel food. There was also little support for an effect of the number of *previous option specific* ingestions by the potential *recipient* (effect size = -0.002; 95% C.I. = -0.02, 0.01) or the potential *donor* (effect size = 0.001; 95% C.I. = -0.01, 0.01). There was also little evidence of an effect of the *age* of the potential *recipient* (effect size = 0.16; 95% C.I. = -0.37, 0.69) *age* of the potential *donor* (effect size = -0.33; 95% C.I. = -1.22, 0.56), or of the *sex* of the potential *recipient* (effect size = -0.03; 95% C.I. = -0.29, 0.23) or *donor* (not present in the top models).

Table S5: GLMM to investigate the effect of food familiarity, individuals’ age and sex, and previous success (continuous) on the probability of success of food transfers (from 233 transfers, of 32 receivers within 6 groups). The table shows the top models, with the number of estimable parameters (Df), AICc values, Δ AIC and Akaike weights (ω_i_) (support for models)

| Model | Df | AICc | Δ AIC | ω_i_ |
| --- | --- | --- | --- | --- |
| Donor Age | 4 | 327.56 | 0.00 | 0.11 |
| Receiver Age | 4 | 327.77 | 0.21 | 0.10 |
| Null | 3 | 327.82 | 0.26 | 0.10 |
| Food Familiarity + Donor Age | 5 | 328.13 | 0.57 | 0.08 |
| Donor Age + Receiver Age | 5 | 328.30 | 0.74 | 0.08 |
| Food Familiarity + Receiver Age | 5 | 328.48 | 0.92 | 0.07 |
| Food Familiarity + Donor Age + Receiver Age | 6 | 328.70 | 1.15 | 0.06 |
| Food Familiarity | 4 | 328.80 | 1.24 | 0.06 |
| Donor Age + Receiver Sex | 5 | 329.20 | 1.64 | 0.05 |
| Donor Option Specific Success + Donor Age | 5 | 329.21 | 1.65 | 0.05 |
| Receiver Sex + Receiver Age | 5 | 329.28 | 1.72 | 0.05 |
| Receiver Sex | 4 | 329.34 | 1.78 | 0.04 |
| Donor State + Receiver Option Specific Success | 5 | 329.35 | 1.79 | 0.04 |
| Food Familiarity + Donor Age + Receiver Option Specific Success | 6 | 329.45 | 1.89 | 0.04 |
| Donor Option Specific Success + Receiver Age | 5 | 329.47 | 1.92 | 0.04 |
| Receiver Option Specific Success | 4 | 329.54 | 1.99 | 0.04 |

Table S6: Table showing the relative importance (sum of Akaike weights), estimates, unconditional standard errors, back-transformed effect on odds of success and their confidence intervals for parameters included in the top models predicting the probability of a success of food transfers

| Variable | Sum of weights | Model averaged estimate (± unconditional SE) | 95% C.I. | Back-transformed effect on odds of success | Back-transformed unconditional 95% C.I. |
| --- | --- | --- | --- | --- | --- |
| Intercept |  | -0.13 (± 0.28) | -0.69, 0.42 | 0.88 baseline odds of success | 0.50, 1.53 |
| Donor Age | 0.51 | -0.33 (± 0.45) | -1.22, 0.56 | 0.72x (juveniles/ adults) | 0.29, 1.75 |
| Receiver Age | 0.39 | 0.16 (± 0.27) | -0.37, 0.69 | 1.17x (juveniles/ adults) | 0.69, 2.00 |
| Food Familiarity | 0.31 | -0.12 (± 0.24) | -0.59, 0.36 | 0.89x (familiar/ novel) | 0.55, 1.43 |
| Receiver Sex | 0.14 | -0.03 (± 0.13) | -0.29, 0.23 | 0.97x (females/ males) | 0.75, 1.26 |
| Receiver Option Specific Success | 0.13 | -0.002 (± 0.008) | -0.02, 0.01 | 1.00x per previous success | 0.98, 1.01 |
| Donor Option Specific Success | 0.09 | 0.001 (± 0.007) | -0.01, 0.01 | 1.00x per previous success | 0.99, 1.01 |

The above results suggest that there is little evidence that food items that were novel before the experiment became less likely to be successfully transferred as the potential donor or recipient ingested more of that food item (i.e. as they became more familiar with it). However, this analysis assumes that the odds of a successful food transfer will be a linear function of the previous number of successes. An alternative possibility is that a single ingestion of a novel food item is enough for a tamarin to become familiar with a food type, and thus decrease the odds of success, without further ingestion events having an effect.

Table S7: GLMM to investigate the effect of food familiarity, individuals’ age and sex, and previous success (binary) on the probability of success of food transfers (from 233 observations, of 32 receivers within 6 groups). The table shows the top models, with the number of estimable parameters (Df), AICc values, Δ AIC and Akaike weights (ω_i_) (support for models)

| Model | Df | AICc | Δ AIC | ω_i_ |
| --- | --- | --- | --- | --- |
| Food Familiarity + Donor Age + Donor Success >0 + Receiver Success >0 | 7 | 321.51 | 0.00 | 0.10 |
| Donor Age + Donor Success >0 + Receiver Success >0 | 6 | 321.52 | 0.02 | 0.10 |
| Food Familiarity + Donor Age + Donor Success >0 | 6 | 321.79 | 0.29 | 0.09 |
| Donor Age + Donor Success >0 | 5 | 321.84 | 0.33 | 0.09 |
| Donor Success >0 | 4 | 322.57 | 1.07 | 0.06 |
| Donor Success >0 + Receiver Success >0 | 5 | 322.59 | 1.08 | 0.06 |
| Food Familiarity + Donor Age + Donor Success >0 + Receiver Sex + Receiver Success >0 | 8 | 322.95 | 1.44 | 0.05 |
| Donor Age + Donor Success >0 + Receiver Sex + Receiver Success >0 | 7 | 323.06 | 1.55 | 0.05 |
| Food Familiarity + Donor Success >0 | 5 | 323.09 | 1.58 | 0.05 |
| Food Familiarity + Donor Age + Donor Success >0 + Receiver Sex | 7 | 323.10 | 1.59 | 0.05 |
| Food Familiarity + Donor Success >0 + Receiver Success >0 | 6 | 323.16 | 1.65 | 0.05 |
| Donor Age + Donor Success >0 + Receiver Sex | 6 | 323.24 | 1.73 | 0.04 |
| Food Familiarity + Donor Age + Donor Success >0 + Receiver Age + Receiver Success >0 | 8 | 323.27 | 1.76 | 0.04 |
| Food Familiarity + Donor Age + Donor Success >0 + Receiver Age | 7 | 323.29 | 1.78 | 0.04 |
| Food Familiarity + Donor Sex + Donor Age + Donor Success >0 + Receiver Success >0 | 8 | 323.36 | 1.85 | 0.04 |
| Donor Age + Donor Success >0 + Receiver Age + Receiver Success >0 | 7 | 323.37 | 1.86 | 0.04 |
| Donor Age + Donor Success >0 + Receiver Age | 6 | 323.45 | 1.94 | 0.04 |

- 1. Influence of donor identity on food transfers

In order to examine whether some donors were more influential than others in the outcome of each analysis, we looked at the random effect for donor identity. In Table S8 we provide the standard deviation (estimate) of the donor identity random effect, its 95 % confidence interval for the top model in each of our reported analysis, and include the table of that analysis, as well as the p-value calculated by comparing the top model which includes the random effects for both donor and receiver identity, and the top model which only includes the receivers’ identity as a random effect. Note that the tables in column three are based on the models within two AIC of the top model, as per the model averaging approach, whereas the values in the other column only refer to the top model, hence we also include in the table the fixed effect that were in the top model (column two).

Table S8: Summary of the effect of donor identity on food transfers. The table shows the model and table the analysis is linked too, as well as the fixed effect in the top model, the standard deviation of the random effect, its 95% confidence interval, p-value, odds ratio corresponding to one standard deviation of the random effect, and the lower and upper limits on the odds ratio

| Model | Fixed effects included in top model | Table | Standard deviation | 95% confidence interval | p-value | Odds ratio | Lower and upper limit of the odds ratio |
| --- | --- | --- | --- | --- | --- | --- | --- |
| Probability of success. Success continuous | Donor age | S6 | 0.334 | 0; 0.859 | 0.475 | 1.40 | 1.00; 2.36 |
| Probability of success. Success binary | Food familiarity + Donor age + Donor success + Receiver success | 2 | 0.303 | 0; 0.850 | 0.570 | 1.35 | 1.00; 2.34 |
| Probability of attempting transfer | Receiver success (binary) | 3 | 0.372 | 0; 0.781 | 0.119 | 1.45 | 1.00; 2.18 |
| Probability of resistance | None | 4 | 1.135 | 0.508; 2.18 | 0.004 | 3.11 | 1.66; 8.84 |

- 1. Probability of attempting a food transfer

Table S9: GLMM to investigate the effect of the food option, individual’s sex, and previous success (binary) on the probability of attempting a food transfer (from 785 observations, of 10 potential receivers within 6 groups). The table shows the top models, with the number of estimable parameters (Df), AICc values, Δ AIC and Akaike weights (ω_i_) (support for models)

| Model | Df | AICc | Δ AIC | ω_i_ |
| --- | --- | --- | --- | --- |
| Receiver Option Specific Success >0 | 4 | 306.29 | 0.00 | 0.50 |
| Receiver Sex + Receiver Option Specific Success >0 | 5 | 307.62 | 1.33 | 0.26 |
| Option + Receiver Option Specific Success >0 | 6 | 307.73 | 1.44 | 0.24 |

- 1. Probability of resistance during a food transfer

Table S10: GLMM to investigate the effect of food familiarity, individuals’ age and sex, and previous success (binary) on the probability of resisting a food transfer (from 116 transfers, of 10 receivers within 6 groups). The table shows the top models, with the number of estimable parameters (Df), AICc values, Δ AIC and Akaike weights (ω_i_) (support for models)

| Model | Df | AICc | Δ AIC | ω_i_ |
| --- | --- | --- | --- | --- |
| Null | 3 | 149.67 | 0.00 | 0.11 |
| Donor Option Specific Success >0 + Receiver Option Specific Success >0 | 5 | 149.94 | 0.26 | 0.10 |
| Receiver Option Specific Success >0 | 4 | 150.30 | 0.63 | 0.08 |
| Donor Option Specific Success >0 | 4 | 150.49 | 0.81 | 0.07 |
| Donor Option Specific Success >0 + Receiver Option Specific Success >0 + Food Option | 7 | 150.51 | 0.84 | 0.07 |
| Food Option | 5 | 150.61 | 0.93 | 0.07 |
| Receiver Sex | 4 | 150.63 | 0.96 | 0.07 |
| Donor Option Specific Success >0 + Receiver Sex + Receiver Option Specific Success >0 + Food Option | 8 | 150.94 | 1.27 | 0.06 |
| Receiver Option Specific Success >0 + Food Option | 6 | 150.95 | 1.28 | 0.06 |
| Donor Option Specific Success >0 + Receiver Sex + Receiver Option Specific Success >0 | 6 | 150.99 | 1.31 | 0.06 |
| Receiver Sex + Food Option | 6 | 151.06 | 1.38 | 0.06 |
| Receiver Sex + Receiver Option Specific Success >0 | 5 | 151.32 | 1.64 | 0.05 |
| Donor Option Specific Success >0 + Food Option | 6 | 151.37 | 1.70 | 0.05 |
| Receiver Sex + Receiver Option Specific Success >0 + Food Option | 7 | 151.43 | 1.75 | 0.05 |
| Donor Option Specific Success >0 + Receiver Sex | 5 | 151.50 | 1.83 | 0.04 |

1. **Results - Third criterion: learning**

Table S11: GLMM to investigate the effect of previous individual eating, social eating (eating food after having obtained it from a food transfer), and exploration of each food type has on the choice of juveniles in the second phase of the experiment (from 7 juveniles within 5 groups). The table shows the top models, with the number of estimable parameters (Df), AICc values, Δ AIC and Akaike weights (ω_i_) (support for models)

| Model | Df | AICc | Δ AIC | ω_i_ |
| --- | --- | --- | --- | --- |
| Exploration + Social Eating | 6 | 197.56 | 0.00 | 0.55 |
| Individual Eating + Social Eating | 6 | 197.93 | 0.36 | 0.45 |
